# Rosuvastatin alters the genetic composition of the human gut microbiome

**DOI:** 10.1038/s41598-020-62261-y

**Published:** 2020-03-25

**Authors:** Martin Kummen, Ole Geir Solberg, Christopher Storm-Larsen, Kristian Holm, Asgrimur Ragnarsson, Marius Trøseid, Beate Vestad, Rita Skårdal, Arne Yndestad, Thor Ueland, Asbjørn Svardal, Rolf K. Berge, Ingebjørg Seljeflot, Lars Gullestad, Tom H. Karlsen, Lars Aaberge, Pål Aukrust, Johannes R. Hov

**Affiliations:** 1 Norwegian PSC Research Center, Department of Transplantation Medicine, Oslo University Hospital Rikshospitalet, Oslo, Norway; 2 Institute of Clinical Medicine, University of Oslo, Oslo, Norway; 3 Research Institute of Internal Medicine, Oslo University Hospital Rikshospitalet, Oslo, Norway; 4 Department of Cardiology, Oslo University Hospital Rikshospitalet, Oslo, Norway; 5 Department of Radiology, Oslo University Hospital, Rikshospitalet, Oslo, Norway; 6 Section of Clinical Immunology and Infectious Diseases, Oslo University Hospital, Oslo, Norway; 7 Center for Heart Failure Research, Oslo University Hospital, Oslo, Norway; 8 K.G. Jebsen Thrombosis Research and Expertise Center, University of Tromsø, Tromsø, Norway; 9 Department of Clinical Science, University of Bergen, Bergen, Norway; 10 Department of Heart Disease, Haukeland University Hospital, Bergen, Norway; 11 Center for Clinical Heart Research, Department of Cardiology, Oslo University Hospital Ullevål, Ullevål, Norway; 12 Section of Gastroenterology, Division of Surgery, Inflammatory Diseases and Transplantation, Oslo University Hospital Rikshospitalet, Oslo, Norway

**Keywords:** Microbiome, Cardiovascular diseases, Drug therapy

## Abstract

The gut microbiome contributes to the variation of blood lipid levels, and secondary bile acids are associated with the effect of statins. Yet, our knowledge of how statins, one of our most common drug groups, affect the human microbiome is scarce. We aimed to characterize the effect of rosuvastatin on gut microbiome composition and inferred genetic content in stool samples from a randomized controlled trial (*n* = 66). No taxa were significantly altered by rosuvastatin during the study. However, rosuvastatin-treated participants showed a reduction in the collective genetic potential to transport and metabolize precursors of the pro-atherogenic metabolite trimethylamine-N-oxide (TMAO, *p* < 0.01), and an increase of related metabolites betaine and γ-butyrobetaine in plasma (*p* < 0.01). Exploratory analyses in the rosuvastatin group showed that participants with the least favorable treatment response (defined as < median change in high-density/low-density lipoprotein (HDL/LDL) ratio) showed a marked increase in TMAO-levels compared to those with a more favorable response (*p* < 0.05). Our data suggest that while rosuvastatin has a limited effect on gut microbiome composition, it could exert broader collective effects on the microbiome relevant to their function, providing a rationale for further studies of the influence of statins on the gut microbiome.

## Introduction

Originally discovered for their anti-microbial properties^1^, statins primarily reduce cholesterol levels and are first-line agents in the management of coronary artery disease (CAD)^2^. The use of statins has been increasing rapidly in recent decades, and they are now one of the most commonly prescribed group of drugs in Western countries^2,3^. The primary mode of action is inhibition of hydroxy-methyl-glutaryl-coenzyme A (HMG-CoA) reductase in the liver leading to decreased levels of low-density lipoprotein (LDL) cholesterol, but statins have other potential beneficial effects *e.g*., anti-inflammatory properties, inhibition of matrix metalloproteinases (MMPs) leading to plaque stabilization and inhibition of platelet aggregation^4^. The mechanisms underlying these pleiotropic effects are not completely understood and may not necessarily be mediated through HMG-CoA reductase inhibition^5–7^.

Changes in the composition and function of gut microbiome have been implicated in the pathogenesis of a wide range of human conditions including various systemic disorders like cardiometabolic and autoimmune disorders^8,9^. In cardiovascular disease (CVD), the gut microbiota-dependent metabolite trimethylamine-N-oxide (TMAO) and related metabolites, has been associated cardiovascular risk^8,9^. The large intra-individual variation of the microbiome could potentially also contribute to variation in both treatment-responses and side effects^10^, and as shown in oncological studies, the microbiome can be used to identify patients responding to treatment^11^. Yet, our knowledge of how non-antibiotic pharmacological agents affect the microbiome and related metabolites is scarce. In cross-sectional studies^12^, and a few randomized controlled trials (*e.g*. metformin and proton pump inhibitors [PPIs]) drugs profoundly affect the gut microbiome and hence potentially its metabolic capacity^13,14^. Interestingly, statins rank second (after PPIs) among drug classes associated with microbiota composition^12^, but so far the ability of statins to modulate the gut microbiome has, to the best of our knowledge, not been tested in randomized controlled trials (RCT).

Some intestinal effects of statins have been observed, including an association with reduced risk of *Clostridioides difficile* (formerly *Clostridium difficile*) infection^6^, and colorectal cancer in patients with inflammatory bowel disease^15^. In addition, in one study, circulating levels of the gut microbial metabolites secondary bile acids predicted the effect on cholesterol profile^7^. Lastly, the gut microbiota has been shown to contribute to a substantial proportion of the variation of blood lipids, especially high-density lipoprotein (HDL)^16^.

In the present study we aimed to investigate the effect of statins on the gut microbiota using stool samples collected from a randomized placebo-controlled double-blinded trial of rosuvastatin treatment in females undergoing coronary angiography for chest pain but with no or minimal CAD (ClinicalTrials.gov-ID: NCT01582165)^17^.

## Patients and Methods

### Trial design and participants

Participants were recruited, and the study was performed as previously described^17^. In short; study participants were recruited at Oslo University Hospital Rikshospitalet, a tertiary care center in Oslo, Norway between June 2012 and December 2015. Female patients aged 30–70 years with suspected ischemic chest pain referred for coronary angiography as part of a diagnostic workup were eligible for inclusion in the study if bicycle ergometry gave positive or equivocal findings.

Overall, 66 participants with normal or near-normal coronary angiograms were included in the study and randomized 1:1 to rosuvastatin 20 mg daily or placebo for 6 months in a double-blinded fashion using a computerized procedure. Stool sampling was part of the study program from included participant number 7.

### Ethics

The study was performed in accordance with Good Clinical Practice and the declaration of Helsinki. Written informed consent was obtained from all study participants prior to heart catheterization. Ethical approval was obtained from Regional Committee for Medical and Health Research Ethics in South-Eastern Norway (reference number 2012/286b & 2011/1600), and the primary study was registered at ClinicalTrials.gov (NCT 01582165) and EUDRACT (2011- 002630-39.3tcAZ).

### Sample collection

Demographic data, medical history and medication were collected at baseline as previously described^17^. Additional dietary data were collected using a questionnaire, as previously described^18^, and participants were instructed not to change diet during the study course. Stool samples were collected at baseline, after four weeks and at study end (Supplementary Fig. S1). All participants used a standardized collection device for stool sampling after voiding^19^, and Stool Collection Tubes with Stool DNA Stabilizer (Stratec Molecular GmbH, Berlin, Germany) was used for transportation by mail to the study center, and samples with >72 h from collection to freezer were excluded (time limit according to the manufacturer). Samples were frozen at −80 °C on arrival. None of the included participants had a history of bowel resection, gastrointestinal stoma or specific diets (*e.g*. vegan, vegetarian, gluten free and milk free diets). If participants reported to have used antibiotics during the four weeks prior to sampling, the sample was collected and sequenced but not included in the analyses. An overview of stool samples not included in the analyses are given in Supplementary Table S1. Peripheral venous blood was collected using pyrogen-free tubes with EDTA as the anticoagulant. Tubes were immediately immersed in melting ice and centrifuged within 30 min at 2000 × g for 20 min to obtain platelet-poor plasma. All samples were stored at −80 °C until being analyzed. Routine blood samples were analyzed using commercial methods. Routine biochemistry was performed at the Department of Medical Biochemistry and extracted from hospital data records. Estimated glomerular filtration rate (eGFR) was calculated according to Levey *et al*.^20^.

### DNA extraction, sequencing and post-processing

DNA was extracted using the PSP Spin Stool DNA Plus Kit (Stratec Molecular GmbH, Berlin, Germany), according to the manufacturer instructions. Library preparations were performed in accordance with a well-established protocol^21^. Libraries were constructed from PCR amplicons of the V3-V4 region of the 16S rRNA gene generated using a unique combination of dual-indexing primers (319F/806R) and Phusion High-Fidelity PCR Master mix with HF buffer (Thermo Fisher Scientific, Waltham, MA, USA). PCR products were normalized using the SequalPrep Normalization Plate Kit (Thermo Fisher Scientific), before pooling and sequencing was performed at the Norwegian Sequencing Centre (Oslo, Norway) on Illumina MiSeq using the v3 kit (San Diego, CA, USA). The Quantitative Insights Into Microbial Ecology (QIIME) pipeline version 1.9.1^22^ was used for post-sequencing processing and closed reference operational taxonomic unit (OTU) mapping to the Silva database (version 128, reference OTUs pre-clustered at 97% sequence similarity) was performed using SortMeRNA version 2.0 through QIIME. OTUs with a number of sequences < 0.005% of the total number of mapped sequences were discarded as recommended^23^, and calculations of rarefied alpha diversity (Chao1 bacterial richness estimate (Chao1), Shannon diversity index and Phylogenetic Diversity) were performed in QIIME The samples were rarefied (subsampled) to an OTU count of 11820 per sample, and all further analyses were performed on the rarefied dataset, and prediction of gut microbial functional profiles based on the 16S rRNA sequencing data were performed using Tax4Fun version 0.3.1^24^.

### Metabolite measurement

Serum levels of betaine, trimethylamine-*N*-oxide (TMAO), carnitine, γ-butyrobetaine and choline were quantified by high performance liquid chromatography as previously described^25^. In summary, we used stable isotope dilution liquid chromatography–tandem mass spectrometry (LC/MS/MS), where we monitored TMAO, choline, betaine, carnitine and γ-butyrobetaine in positive liquid chromatography–tandem mass spectrometry (MRM) MS mode and the characteristic precursor–product ion transitions: *m/z* 76→58, *m/z* 104→60, *m/z* 118→58, *m/z* 162→103 and *m/z* 146→87, respectively, was used. Before protein precipitation, internal standards were added to the plasma samples. The internal standards (TMAO-trimethyl-d9 (d9-TMAO), choline-trimethyl-d9 (d9-choline), L-carnitine-d3 (methyl-d3), betaine-trimethyl-d9-methylene-d2 (d11-betaine) and γ-butyrobetaine N-(carboxypropyl)-N,N,N,trimethyl-d9) were similarly monitored in MRM mode at *m/z* 85→66, *m/z* 113→69, *m/z* 165→103 and *m/z* 129→66 and *m/z* 155→87, respectively. To prepare the calibration curves for the quantification of plasma analyte, various concentrations of carnitine, betaine, TMAO, choline and γ-butyrobetaine standards and a fixed amount of internal standards was spiked into 4% bovine serum albumin^25^. All the stable isotope-labelled internal standards were purchased from Cambridge Isotope Laboratories, Inc. (Andover, MA, USA) except for carnitine and γ-butyrobetaine which were purchased from CDNI Isotopes (Quebec, Canada).

### Power estimates

We had 80% power to detect a ≥ 50% change in relative abundance of bacterial taxa at the genus level of ≥0.5% mean relative abundance, and a ≥ 16% change in alpha diversity, caused by rosuvastatin.

### Statistical analysis

Comparison of categorical variables was performed using the Chi-square test. Distribution of continuous variables were evaluated using histograms and Students t-test and the Mann-Whitney U test was used as appropriate. The Mann-Whitney U test was used to compare the differences in the change of taxa on the genus level and KEGG Orthologs between study groups in the initial screening, and these calculations were performed in R version 3.4.1. Paired samples were subsequently compared using a general linear model ANOVA with repeated measures in SPSS Statistics for Macintosh, version 25 (IBM, Armonk, NY, USA) and *p*-values denoted *P*_GLM_, and non-normalized values were log-transformed before these analyses. For correlation analyses, the Spearman’s rank correlation test was utilized. False-discovery rate (FDR) was calculated according to Benjamini-Hochberg using R. FDR-corrected p-values were denoted Q_FDR_. Unless otherwise stated, all other calculations were performed in SPSS.

## Results

After exclusions for antibiotics use, failure to deliver adequate sample and post-sequencing quality control (Supplementary Table S1), microbiota data and plasma samples from baseline and study end were available from 40 and 51 individuals, respectively (microbiota data: *n* = 20 in both the placebo and the rosuvastatin group; plasma: *n* = 25 in the placebo group and *n* = 26 in the rosuvastatin group). Demographics for randomized participants with microbiota data available are given in Table 1. Randomized participants with and without microbiota data available did not differ at baseline (Supplementary Table S2).

**Table 1:** Baseline characteristics of randomized patients with microbiota data available from baseline and study end.

	Placebo	Rosuvastatin	*p* value	*n*^a^
	*n =* 20	*n =* 20		
Age, years	51.5 (±8.7)	59.3 (±6.9)	0.003	
Sex (female)	20 (100)	20 (100)	1.000	
Current or former smoker	13 (65)	13 (65)	1.000	
Body mass index, kg/m^2^	28.3 (±5.1)	25.2 (±3.8)	0.052^b^	
Hypertension	4 (20)	5 (25)	0.500	
Diabetes mellitus type 2	0 (0)	0 (0)	—	
Family history of CAD	16 (80)	13 (65)	0.240	
*Medication*				
ACEi/ARB	3 (15)	1 (5)	0.302	
Beta blockers	8 (40)	5 (25)	0.250	
Calcium-channel blocker	1 (5)	1 (5)	0.756	
Aspirin	14 (70)	13 (65)	0.500	
Proton pump inhibitors	3 (15)	0 (0)	0.115	
*Biochemistry*				
Total cholesterol, mmol/L	6.0 (±1.5)	5.9 (±1.0)	0.923	
LDL, mmol/L	4.0 (±1.4)	3.7 (±0.9)	0.422	
HDL, mmol/L	1.5 (±0.6)	1.9 (±0.4)	0.035	
Triglycerides, mmol/L	1.8 (±0.9)	1.3 (±0.6)	0.038	
Hemoglobin, g/dL	14.1 (±0.7)	13.9 (±0.8)	0.485	
Creatinine, µmol/L	65.5 (±11.4)	64.6 (±7.0)	0.765	
Total bilirubin, mg/dL	6.3 (±2.4)	7.5 (±5.0)	0.362	18/19
AST, U/L	22.4 (±6.7)	25.5 (±5.9)	0.134	
ALT, U/L	21.1 (±11.3)	22.4 (±7.5)	0.674	19/20
ALP, U/L	65.6 (±18.5)	62.0 (11.7)	0.481	17/19
HbA1c, %	5.7 (±0.4)	5.6 (±0.4)	0.510	
CRP, mg/L	4.0 (±3.7)	2.3 (±2.1)	0.099^b^	

Data are mean ± SD or n (%) values. ^a^Complete data unless specified as n in placebo/rosuvastatin. ^b^Right-skewed data compared using the Mann-Whitney U test, all other variables compared using the Students t-test. ACEi, angiotensin-converting-enzyme inhibitor; ALP, alkaline phosphatase; ALT, alanine transaminase, ARB, angiotensin II receptor blockers; AST, aspartate transaminase; CAD, coronary artery disease; CRP, C-reactive protein; HDL, high-density lipoprotein; LDL, low-density lipoprotein.

**Figure 1:**
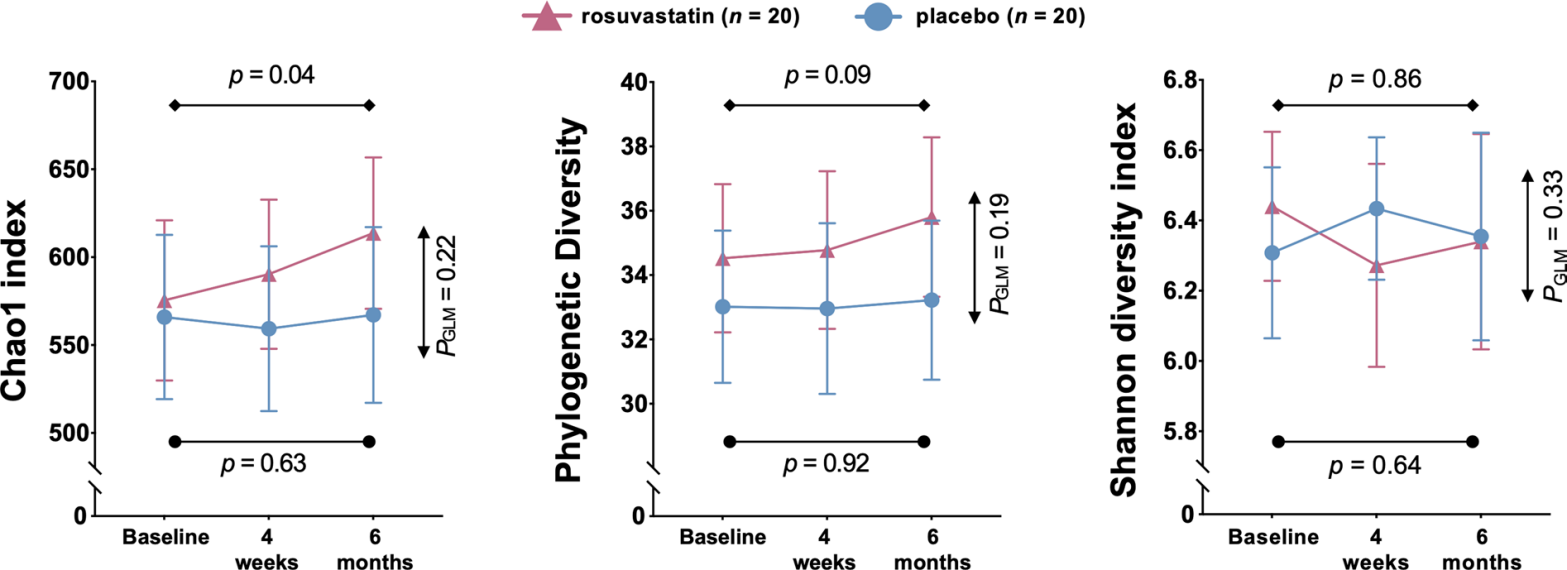
A trend towards increased gut microbial richness in participants receiving rosuvastatin. Participants on rosuvastatin treatment showed an increase in gut microbial richness (delta Chao1, *paired t-test p =* 0.04), but this increase was not statistically significant compared with the placebo group (*P*_GLM_ = 0.22). A similar trend was detected for Phylogenetic diversity, while the change in Shannon diversity index was similar in the rosuvastatin and the placebo group. Data shown as mean ±95% CI. Paired t-test from baseline and study-end within the same study group, denoted *p*. Comparison of change between the study groups using repeated measures ANOVA from baseline and study-end, denoted *P*_GLM_. Values at 4 weeks missing for *n* = 1 in each group.

Participants on rosuvastatin treatment showed an increase in gut microbial richness, but not statistically significant compared with placebo (delta Chao1 8.6 [95% CI −27.7−44.8], *P*_GLM_ = 0.22, Fig. 1). The treatment group did not show changes in other diversity measurements either, *i.e*. Phylogenetic Diversity and Chao1, compared to the placebo group (Fig. 1).

Compared with changes in the placebo-group, rosuvastatin had a very limited effect on the relative abundance of bacterial taxa at the genus level over the study period (Supplementary Table S3). Out of the 173 genera detected at baseline, 38 (22.0%) had a mean relative abundance ≥0.5%.

**Figure 2:**
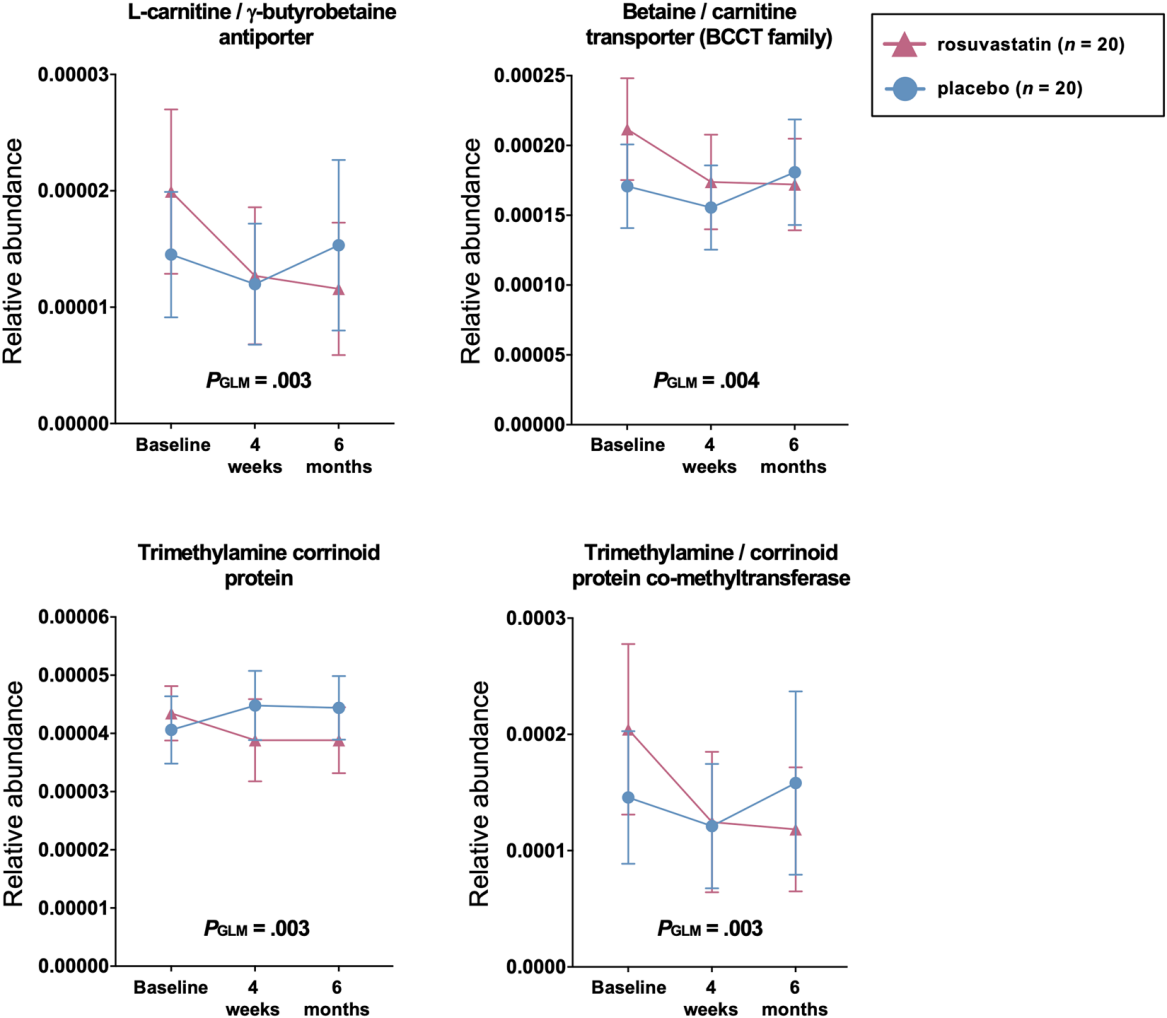
Functions (KEGG Orthologs) in the gut microbiota related to cellular transport and metabolism along the choline/betaine-trimethylamine (TMA) metabolic pathway are affected by rosuvastatin treatment. Data are shown as mean ±95% CI. Repeated measures ANOVA from baseline and study-end, denoted *P*_GLM_. Values at four weeks missing for *n =* 1 in each group.

Despite the lack of significant compositional changes at the genus level, pharmacological treatment may induce more broad changes to several different taxonomic groups that share similar function. To investigate whether rosuvastatin affected the functional potential of the gut microbiota, we inferred microbial gene content in the samples based on the 16 S rRNA sequencing data. Out of the top 20 altered gene functions, the majority were un-related KEGG orthologs, however, four out of the 20 were related to cellular transport and metabolism along the choline/betaine-TMA metabolic pathway (*P* < 0.005, Q_FDR_ < 0.35, Supplementary Table S4), all showing a reduction in the rosuvastatin group compared to placebo (Fig. 2**)**. Furthermore, among all inferred genes we identified four additional functions related to the same pathway that were significantly altered, all showing reduction on rosuvastatin treatment (Supplementary Fig. S2).

**Figure 3:**
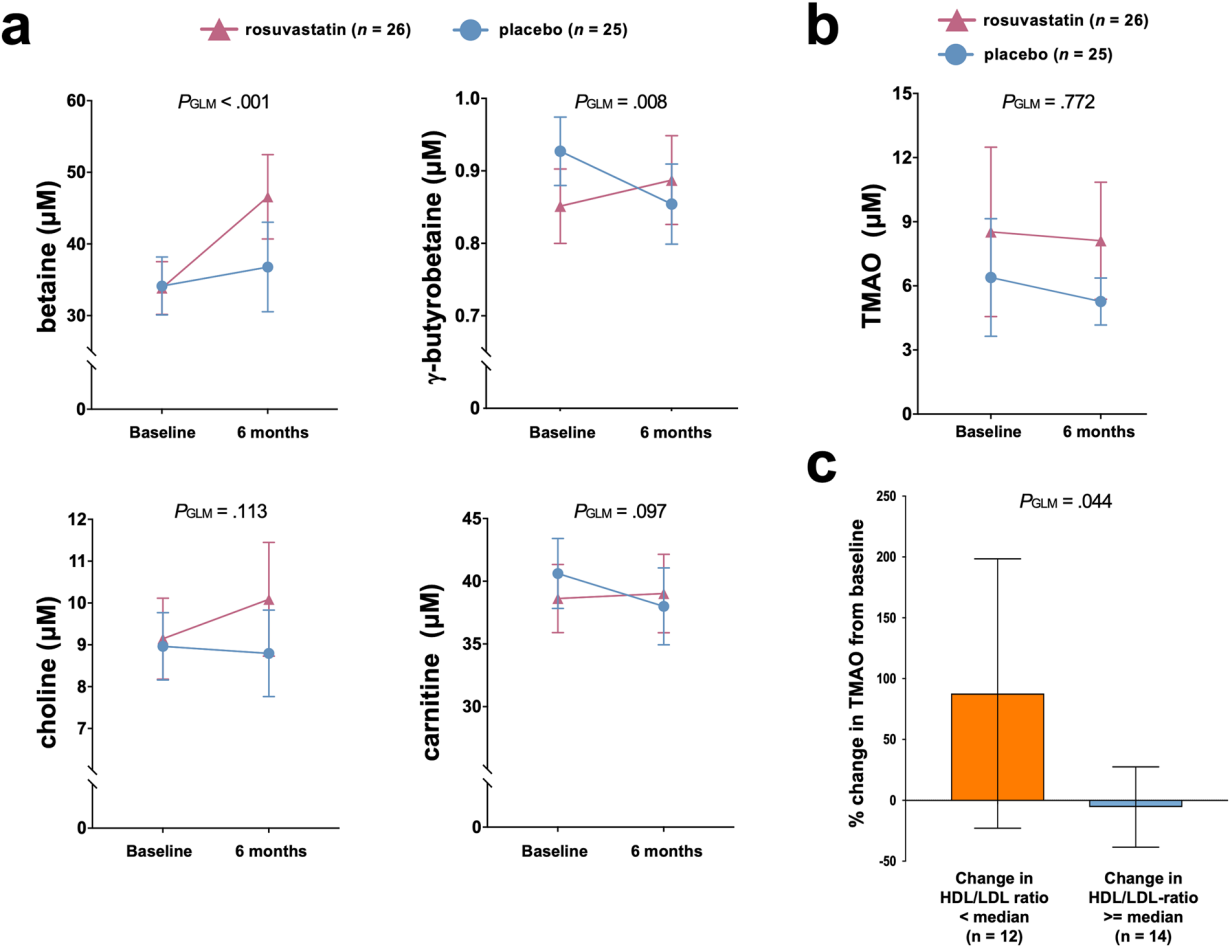
Changes in microbiome-related metabolites in peripheral blood during rosuvastatin treatment. (**a**) Rosuvastatin increase precursors of the microbiota dependent metabolite trimethylamine (TMA) in plasma. (**b**) TMA is metabolized to the pro-atherogenic metabolite trimethylamine-N-oxide (TMAO) in the liver, which is not affected by rosuvastatin. (**c**) Participants with the least favorable treatment response (defined as below median change in high density to low density lipoprotein (HDL/LDL) ratio) show a marked increase in TMAO levels compared to those with a more favorable response. All randomized participants with serum samples available were included in the analysis, irrespective of the availability of microbiome data. TMAO values missing for *n =* 2 in panel C. Data shown as mean ±95% CI. Repeated measures ANOVA, denoted *P*_GLM_.

We hypothesized that alterations in microbial functions and metabolites in the gut would be accompanied by corresponding changes in peripheral blood. We therefore measured metabolites in plasma by liquid chromatography-tandem mass spectrometry. Betaine and γ-butyrobetaine, both metabolites related to the phosphatidylcholine/carnitine-TMA-TMAO pathway increased significantly in the rosuvastatin group compared to the placebo group (Fig. 3a, both *P*_GLM_ < 0.01). Both carnitine and choline showed a trend towards an increase, but did not reach the significance threshold (Fig. 3a). However, TMAO was unaffected (Fig. 3b), also when adjusting for renal function (using eGFR, data not shown). There was no significant change in renal function in either of the study groups during follow-up (data not shown). Repeating the analyses with only those participants with microbiota-data available caused only minor changes: the increase of carnitine in the rosuvastatin group (*n* = 17) was significant compared to placebo (*n* = 16, *P*_GLM_ = 0.044), while the results for betaine, γ-butyrobetaine and choline were similar (*P*_GLM_ = 0.01, 0.007 and 0.084, respectively).

The relative abundance of the inferred genes shown in Fig. 2 did not correlate with plasma levels of the measured metabolites at baseline (data not shown). Changes occurring in plasma levels of betaine and γ-butyrobetaine during follow-up in the placebo group correlated with the change in the relative abundance of the genes encoding for both glycine betaine transporter (rho = 0.58, *p* = 0.014 and rho = 0.54, *p* = 0.025, respectively) and trimethylamine-corrinoid protein co-methyltransferase (rho = 0.60, *p* = 0.011 and rho = 0.53, *p* = 0.029, respectively) (Supplementary Table S5). In contrast, there were no significant correlations between changes in plasma and microbial genes in the rosuvastatin group (all *p* > 0.35, Supplementary Table S5).

It is well known that the individual response to statin treatment shows large variations^7^. As statins primarily reduce low density lipoprotein (LDL) levels and the gut microbiota especially contribute to variation in HDL levels^16^, we used HDL to LDL ratio to evaluated treatment response, when performing exploratory analysis in the rosuvastatin group. Participants with a poor treatment response (defined as below the median change in HDL to LDL ratio) showed a significant increase in TMAO values, compared to the other participants (Fig. 3c).

## Discussion

To our knowledge, this is the first study investigating the effects of a statin on the human gut microbiota using samples from a randomized controlled trial. We found that rosuvastatin in general had small effects on gut microbial composition. On the other hand, rosuvastatin reduced the genetic potential of the gut microbiota to metabolize and transport several molecules along the choline/betaine-TMA metabolic pathway, with corresponding changes of related metabolites in plasma. Finally, although rosuvastatin did not induce changes in TMAO levels in the treatment group as a whole, those who had a poor improvement in HDL/LDL ratio had an increase in this pro-atherogenic gut-microbiota-derived metabolite indicating some relationship between statin effects and the gut microbiome.

Little is known about the direct effect of statins on the human gut microbiota, except for associations with overall bacterial composition in a population-based study^12^, and some differences between patients with and without normalization of blood lipid levels^26^. Herein, rosuvastatin treatment was associated with a trend towards increasing gut microbial richness, as reported in studies of atorvastatin-treated rats^5^, while studies in mice have been unable to detect changes in fecal microbial diversity after rosuvastatin treatment^27,28^. In contrast to previous association studies in humans, our randomized placebo-controlled trial showed in general only modest and non-significant effects of statin intervention (*i.e*., rosuvastatin) on gut microbiome composition. Studies in mice have shown a similar very modest impact of rosuvastatin on fecal bacterial composition^27,28^, and at physiologically relevant concentrations rosuvastatin has little impact on bacterial growth *in vitro*^28^. However, the rather small sample size of our study makes it under-powered to detect subtle changes in relative abundance in general and also for broader changes in low-abundant taxa.

Microbial influence on disease is perhaps more likely to relate to the collective function of the microbiome, rather than composition^12^. Whereas the overall effects of rosuvastatin on gut microbiome composition was poor, by inferring genetic content, we observed a reduction of several microbial genes encoding proteins related to the choline/betaine-TMA-pathway (overview in Supplementary Fig. S3). This could suggest that rosuvastatin interferes with the microbiome’s ability to metabolize betaine and related metabolites, with corresponding changes in levels of metabolites in plasma, as we also observed. Of the measured metabolites in our study rosuvastatin had the greatest effect on betaine, which has previously been shown to inversely correlate with both triglycerides and non-HDL cholesterol^29^, and alleviate inflammation by lowering interleukin (IL)-1β secretion^30^. Increased betaine has also been associated with a reduced risk of both CVD and stroke^31^. The direct relationship between changes in plasma and gut during follow-up was however not immediately intuitive, since there was a correlation between changes in affected microbial genes and changes in metabolites during follow-up only in the placebo group. Although one should interpret result from such subgroup analyses with caution, it could be speculated that rosuvastatin somehow disrupts the steady-state of intestinal metabolism and/or absorption of these compounds. It is also possible that the increase could in part be explained by a more direct effect of statins on endogenous human sources^31,32^. However, bacterial and human metabolisms of these metabolites are intricate and far from completely understood^33–35^, as also illustrated in Supplementary Fig. S3, and experimental studies are needed to elucidate this further^36^.

Overall, however, rosuvastatin did not reduce TMAO in this study, but importantly, in the group with the least favorable effect of rosuvastatin on HDL/LDL-ratio, TMAO significantly increased during the study. The individual response to statin treatment in large clinical trials show large variations, and the number needed to treat (NNT) for rosuvastatin in women is 31 (*i.e*., one has to treat 31 women with rosuvastatin to prevent the first cardiovascular event or death)^37^. The gut microbiome also show large variation between people in the general population^10,38^, and probably contribute to the variation in treatment response of pharmacological agents, as recently show in several studies^39–41^, both directly my affecting drug metabolism^36^, through interaction with the immune system^11^, and possibly also effects on human metabolism. Taken together, it is tempting to hypothesize that this may contribute to some of the large variation in statin treatment response and pleiotropic effects of statins, as *e.g*., anti-inflammatory properties, stabilization of atherosclerotic plaques and inhibition of platelet aggregation, as these are all associated with the betaine/choline-TMA-TMAO pathway^8,9^. Re-analyzing TMAO-related metabolites in material from previous statin-trials could be a logical next step.

In contrast to these observations, we were unable to detect any changes in different taxonomic groups induced by rosuvastatin treatment. This is a little surprising, given that statins in a recent large population-based cohort from Europe was the drugs with the second strongest association with over-all bacterial composition (measured by ﻿Bray-Curtis distance), somewhat less than PPI, but stronger than the association with antibiotics, although the explained variance of statin use on over-all bacterial composition in this study was only ~0.3%^12^. That rosuvastatin show effect in the abundance of several genes, while not affecting abundance of individual taxa might seem contradictive, but could be explained by more subtle effect across multiple taxa that share the same genes. Our data could indicate that rosuvastatin affected the abundance of a genus in the Ruminococcaceae-family, which has previously been associated with the response to statin treatment^26^, but this change was not significant after adjusting for multiple testing.

Our study has some strengths and limitations that warrant further discussion. The randomized controlled study design is a major strength, as is the performance of coronary angiography in all participants. Still, independent validation is necessary before a conclusion can be made. The main limitation of our study is the modest sample size with the corresponding loss of power. In addition, as in most microbiome studies there are some concerns regarding multiple testing, which to some degree can be improved by adjusting p-values, as we did, although the ideal would be replication in separate cohorts. Dietary data were collected at baseline, but no in-depth survey (*e.g*. food-frequency or recall questionnaires) and all participants were instructed to avoid dietary changes during the study and none reported specific diets. Furthermore, inferring genetic content from 16S rRNA data has some limitations compared to shotgun sequencing although the correlation between these methods for human gastrointestinal samples is high (~0.85)^24^.

## Conclusion

We report data from a randomized placebo-controlled double-blinded trial of rosuvastatin treatment showing a minor effect of rosuvastatin on taxonomic composition of the gut microbiome. On the other hand, the collective genetic potential of the gut microbiome to transport and metabolize metabolites along the phosphatidylcholine/carnitine-TMA-TMAO pathway was reduced in the rosuvastatin treated group, with a corresponding increase of several metabolites in plasma. Our findings suggest that statins could have a clinically relevant impact on the gut microbiome and provide a strong rationale for further and larger studies of the influence of statins on the gut microbiome, TMAO and related metabolites in the progression of CVD, and its influence on intra-individual treatment responses.

## Supplementary information

Supplementary Information.
