# Supplementary material for: Rosuvastatin alters the genetic composition of the human gut microbiome
Source: Sci Rep. 2020 Mar 25;10:5397. doi: 10.1038/s41598-020-62261-y (PMC7096534; doi:10.1038/s41598-020-62261-y)
Supplement: Supplementary file 1 — Supplementary Information. [file 41598_2020_62261_MOESM1_ESM.pdf]

SUPPLEMENTAL MATERIAL

Rosuvastatin alters the genetic composition of the human gut microbiome

Martin Kummen[1,2,3], Ole Geir Solberg[4], Christopher Storm-Larsen[1,2,3], Kristian Holm[1,2,3], Asgrimur Ragnarsson[5], Marius Trøseid[2,3,6], Beate Vestad[2,3], Rita Skårdal[4], Arne Yndestad[2,3,7], Thor Ueland[2,3,8], Asbjørn Svardal[9], Rolf K. Berge[9,10], Ingebjørg Seljeflot[2,11], Lars Gullestad[2,4], Tom H. Karlsen[1,2,3,12], Lars Aaberge[4], Pål Aukrust[2,3,6], Johannes R. Hov[1,2,3,12].

[1]Norwegian PSC Research Center, Department of Transplantation Medicine, Oslo University Hospital Rikshospitalet, Oslo, Norway; [2]Institute of Clinical Medicine, University of Oslo, Oslo, Norway; [3]Research Institute of Internal Medicine, Oslo University Hospital Rikshospitalet, Oslo, Norway; [4]Department of Cardiology, Oslo University Hospital Rikshospitalet, Oslo, Norway; [5]Department of Radiology, Oslo University Hospital, Rikshospitalet, Oslo, Norway; [6]Section of Clinical Immunology and Infectious Diseases, Oslo University Hospital, Oslo, Norway; [7]Center for Heart Failure Research, Oslo University Hospital, Oslo, Norway; [8]K.G. Jebsen Thrombosis Research and Expertise Center, University of Tromsø, Tromsø, Norway; [9]Department of Clinical Science, University of Bergen, Bergen, Norway; [10]Department of Heart Disease, Haukeland University Hospital, Bergen, Norway; [11]Center for Clinical Heart Research, Department of Cardiology, Oslo University Hospital Ullevål, Norway; [12]Section of Gastroenterology, Division of Surgery, Inflammatory Diseases and Transplantation, Oslo University Hospital Rikshospitalet, Oslo, Norway.

Table of Contents

Supplementary Tables **3**

Supplementary Table S1 .... *3*

Supplementary Table S2 .... *4*

Supplementary Table S3 .... *5*

Supplementary Table S4 .... *8*

Supplementary Table S5 .... *9*

Supplementary Figures **10**

Supplementary Fig. S1 .... *10*

Supplementary Fig. S2 .... *11*

Supplementary Fig. S3 .... *12*

Supplementary References **13**

Supplementary Tables

Supplementary Table S1

Supplementary Table S1. Missing microbiota samples during follow-up and exclusions due to technical issues.

	**Placebo**		**Rosuvastatin**	
Randomized patients at baseline	n = 33 (100%)		n = 33 (100%)	
No microbiota samples available at any time point#	8	(24.2)	9	(27.3)
**Reason for sample exclusion - baseline samples**				
Participant did not deliver stool sample	-		-	
Antibiotics within the last 4 weeks	1	(3.0)	-	
Samples in room temperature >72 hours	-		1	(3.0)
Failed sequencing	-		1	(3.0)
**Reason for sample exclusion - 4 weeks follow-up**				
Participant did not deliver stool sample	2*	(6.1)	2*	(6.1)
Antibiotics within the last 4 weeks	1*	(3.0)	-	
Samples in room temperature >72 hours	-		-	
Failed sequencing	-		-	
**Reason for sample exclusion - 6 months follow-up**				
Participant did not deliver stool sample	2*	(6.1)	2*	(6.1)
Antibiotics within the last 4 weeks	2*	(6.1)	-	
Samples in room temperature >72 hours	-		-	
Failed sequencing	-		-	
Stool sample available for ≥2 time points	23	(69.7)	23	(69.7)
Stool sample available from baseline and 6 months	20	(60.6)	20	(60.6)

#Stool sampling was part of the study program from participant number 7.
*Samples from the same individual were excluded for the same reason at both 4 week and 6 months follow-up (2 individuals in the placebo group, 1 individual in the rosuvastatin group).

Supplementary Table S2

Supplementary Table S2. Baseline characteristics of the included patients with available microbiota samples compared with those without. Data presented as mean±SD or n (%).

	Randomized participants (n = 66)			
	Microbiota available for ≥2 time points	Microbiota available for <2 time points		
	n = 46 (69.7)	n = 20 (30.3)	*P* value	*n**
Age, years	55.3 (±8.9)	51.2 (±9.7)	0.107	
Sex (female)	46 (100)	20 (100)		
Current or former smoker	29 (63.0)	15 (75.0)	0.405	
Body mass index, kg/m^2	26.7 (±4.7)	25.0 (±3.6)	0.299#	
Hypertension	11 (23.9)	4 (20.0)	0.765	
Diabetes mellitus type 2	0 (0)	2 (10.0)	0.089	
Family history of CAD	35 (76.1)	18 (90.0)	0.314	
Medication				
ACEi/ARB	4 (8.7)	4 (20.0)	0.232	
Beta blockers	13 (28.3)	7 (35.0)	0.771	
Calcium-channel blocker	4 (8.7)	2 (10.0)	1.000	
Aspirin	30 (65.2)	14 (70.0)	0.782	
Proton pump inhibitors	4 (8.7)	2 (10.0)	1.000	
Biochemistry				
Total cholesterol, mmol/L	5.8 (±1.2)	5.7 (±1.0)	0.619	
LDL-C, mmol/L	3.7 (±1.1)	3.6 (±0.9)	0.521	
HDL-C, mmol/L	1.7 (±0.5)	1.6 (±0.4)	0.774	
Triglycerides, mmol/L	1.5 (±0.8)	1.4 (±0.6)	0.673	
Hemoglobin, g/dL	14.0 (±0.8)	13.7 (±1.0)	0.265	
Creatinine, µmol/L	65.3 (±8.9)	61.6 (±9.1)	0.126	
Total bilirubin, mg/dL	6.9 (±3.7)	5.8 (±1.6)	0.252	(43/19)
AST, U/L	23.7 (±6.3)	23.8 (±5.7)	0.984	
ALT, U/L	21.1 (±9.2)	21.0 (±9.0)	0.957	(45/20)
ALP, U/L	64.0 (±14.7)	69.2 (±30.1)	0.369	(42/18)
HbA1c, %	5.6 (±0.4)	5.7 (±0.5)	0.444	
CRP, mg/L	2.9 (±2.3)	1.9 (±1.8)	0.164#	
Carnitine metabolites				
Betaine, µM	33.3 (±10.2)	33.0 (±9.2)	0.893	(45/19)
Carnitine, µM	38.4 (±7.2)	38.8 (±6.6)	0.832	(45/19)
Choline, µM	9.0 (±2.2	8.4 (±1.9)	0.304	(45/19)
γ-butyrobetaine, µM	0.9 (±0.1)	0.9 (±0.2)	0.658	(45/19)
Trimethylamine-*N*-oxide, µM	8.0 (±9.9)	6.3 (±7.6)	0.164#	(45/19)

*Complete data unless otherwise specified. #Right-skewed data compared using the Mann-Whitney U test, all other variables compared using the Students t-test. ACEi, angiotensin-converting-enzyme inhibitor; ALP, alkaline phosphatase; ALT, alanine transaminase, ARB, angiotensin II receptor blockers; AST, aspartate transaminase; CAD, coronary artery disease; CRP, C-reactive protein; HDL, high-density lipoprotein; LDL, low-density lipoprotein.

Supplementary Table S3

Supplementary Table S3. Changes in bacterial taxa from baseline to study end (6 months) at the genus level.

Taxonomy	Median change in relative abundance from baseline			
	rosuvastatin	placebo	*P* value*	Q_{FDR}
Firmicutes - Clostridia - Clostridiales - Ruminococcaceae - Ruminococcaceae UCG-002	-2.572E-02	-2.369E-03	0.003	0.54
Firmicutes - Clostridia - Clostridiales - Lachnospiraceae - [Eubacterium] hallii group	0	1.692E-04	0.047	1
Firmicutes - Clostridia - Clostridiales - Lachnospiraceae - Hungatella	-8.460E-05	0	0.064	1
Actinobacteria - Coriobacteriia - Coriobacteriales - Coriobacteriaceae - Eggerthella	0	0	0.072	1
Firmicutes - Clostridia - Clostridiales - Lachnospiraceae - [Bacteroides] pectinophilus group	0	0	0.085	1
Firmicutes - Clostridia - Clostridiales - Ruminococcaceae - [Eubacterium] coprostanoligenes group	-1.354E-03	1.692E-04	0.098	1
Firmicutes - Clostridia - Clostridiales - Ruminococcaceae - Ruminococcaceae UCG-009	0	0	0.100	1
Firmicutes - Clostridia - Clostridiales - Lachnospiraceae - Lachnospiraceae UCG-004	2.538E-04	-3.384E-04	0.110	1
Firmicutes - Clostridia - Clostridiales - Peptostreptococcaceae - Peptoclostridium	0	1.692E-04	0.112	1
Firmicutes - Erysipelotrichia - Erysipelotrichales - Erysipelotrichaceae - uncultured	0	0	0.123	1
Bacteroidetes - Bacteroidia - Bacteroidales - Porphyromonadaceae - Odoribacter	-7.614E-04	0	0.123	1
Firmicutes - Clostridia - Clostridiales - Lachnospiraceae - uncultured	2.538E-04	2.453E-03	0.130	1
Bacteroidetes - Bacteroidia - Bacteroidales - Porphyromonadaceae - Butyricimonas	-8.460E-05	0	0.133	1
Firmicutes - Clostridia - Clostridiales - Lachnospiraceae - Lachnospiraceae UCG-010	0	8.460E-05	0.143	1
Firmicutes - Clostridia - Clostridiales - Lachnospiraceae - Mobilitalea	0	0	0.143	1
Tenericutes - Mollicutes - Anaeroplasmatales - Anaeroplasmataceae - Anaeroplasma	0	0	0.143	1
Bacteroidetes - Flavobacteriia - Flavobacteriales - Flavobacteriaceae - uncultured	0	0	0.147	1
Firmicutes - Clostridia - Clostridiales - Clostridiales vadinBB60 group - uncultured organism	0	0	0.154	1
Proteobacteria - Betaproteobacteria - Burkholderiales - Alcaligenaceae - Parasutterella	0	0	0.159	1
Bacteroidetes - Bacteroidia - Bacteroidales - Prevotellaceae - Alloprevotella	0	0	0.172	1
Firmicutes - Erysipelotrichia - Erysipelotrichales - Erysipelotrichaceae - Catenibacterium	0	0	0.172	1
Actinobacteria - Coriobacteriia - Coriobacteriales - Coriobacteriaceae - Collinsella	0	-1.692E-04	0.190	1
Actinobacteria - Coriobacteriia - Coriobacteriales - Coriobacteriaceae - Gordonibacter	0	0	0.192	1
Firmicutes - Negativicutes - Selenomonadales - Veillonellaceae - Dialister	0	0	0.206	1
Bacteroidetes - Bacteroidia - Bacteroidales - Porphyromonadaceae - Coprobacter	0	0	0.211	1
Firmicutes - Clostridia - Clostridiales - Lachnospiraceae - Lachnospiraceae UCG-008	1.946E-03	3.976E-03	0.222	1
Firmicutes - Clostridia - Clostridiales - Lachnospiraceae - Anaerostipes	-8.460E-05	0	0.234	1
Actinobacteria - Coriobacteriia - Coriobacteriales - Coriobacteriaceae - Senegalimassilia	0	0	0.238	1
Firmicutes - Erysipelotrichia - Erysipelotrichales - Erysipelotrichaceae - Dielma	0	0	0.238	1
Firmicutes - Clostridia - Clostridiales - Lachnospiraceae - Coprococcus 3	1.692E-04	-8.460E-05	0.243	1
Firmicutes - Clostridia - Clostridiales - Ruminococcaceae - Ruminiclostridium 5	-2.538E-04	2.538E-04	0.245	1
Bacteroidetes - Bacteroidia - Bacteroidales - Bacteroidaceae - Bacteroides	-1.294E-02	1.506E-02	0.259	1
Firmicutes - Erysipelotrichia - Erysipelotrichales - Erysipelotrichaceae - Erysipelotrichaceae UCG-003	-8.460E-05	0	0.262	1
Firmicutes - Negativicutes - Selenomonadales - Acidaminococcaceae - Acidaminococcus	0	0	0.285	1
Firmicutes - Clostridia - Clostridiales - Lachnospiraceae - uncultured bacterium	8.460E-05	0	0.306	1
Bacteroidetes - Bacteroidia - Bacteroidales - Prevotellaceae - Prevotella 2	0	0	0.317	1
Bacteroidetes - Bacteroidia - Bacteroidales - Prevotellaceae - Prevotellaceae UCG-003	0	0	0.317	1
Bacteroidetes - Bacteroidia - Bacteroidales - Prevotellaceae - uncultured	0	0	0.317	1
Firmicutes - Clostridia - Clostridiales - Peptostreptococcaceae - Terrisporobacter	0	0	0.317	1
Firmicutes - Negativicutes - Selenomonadales - Veillonellaceae - Mitsuokella	0	0	0.317	1
Proteobacteria - Gammaproteobacteria - Enterobacteriales - Enterobacteriaceae - Klebsiella	0	0	0.317	1
Firmicutes - Clostridia - Clostridiales - Lachnospiraceae - Howardella	0	0	0.317	1
Actinobacteria - Coriobacteriia - Coriobacteriales - Coriobacteriaceae - Enterorhabdus	0	0	0.319	1
Firmicutes - Clostridia - Clostridiales - Ruminococcaceae - Ruminococcaceae NK4A214 group	8.460E-05	-1.777E-03	0.324	1
Bacteroidetes - Bacteroidia - Bacteroidales - Bacteroidales S24-7 group - uncultured bacterium	0	1.692E-04	0.329	1
Firmicutes - Negativicutes - Selenomonadales - Veillonellaceae - Veillonella	0	0	0.336	1
Firmicutes - Erysipelotrichia - Erysipelotrichales - Erysipelotrichaceae - Turicibacter	0	0	0.347	1
Bacteroidetes - Bacteroidia - Bacteroidales - uncultured - uncultured bacterium	0	0	0.363	1
Cyanobacteria - Melainabacteria - Gastranaerophilales - uncultured organism - uncultured organism	0	0	0.363	1
Firmicutes - Clostridia - Clostridiales - Peptococcaceae - Peptococcus	0	0	0.363	1
Firmicutes - Negativicutes - Selenomonadales - Veillonellaceae - Megasphaera	0	0	0.363	1
Proteobacteria - Gammaproteobacteria - Alteromonadales - Shewanellaceae - Shewanella	0	0	0.363	1
Firmicutes - Clostridia - Clostridiales - Ruminococcaceae - Flavonifractor	-1.692E-04	-8.460E-05	0.365	1
Firmicutes - Clostridia - Clostridiales - Ruminococcaceae - Oscillospira	0	0	0.368	1
Firmicutes - Clostridia - Clostridiales - Ruminococcaceae - Oscillibacter	1.692E-04	1.692E-04	0.372	1
Firmicutes - Clostridia - Clostridiales - Lachnospiraceae - Coprococcus 2	0	0	0.374	1
Firmicutes - Clostridia - Clostridiales - Lachnospiraceae;Ambiguous_taxa	4.230E-04	1.692E-04	0.378	1
Cyanobacteria - Melainabacteria - Gastranaerophilales - uncultured rumen bacterium - uncultured rumen bacterium	0	0	0.383	1
Firmicutes - Clostridia - Clostridiales - Ruminococcaceae;Ambiguous_taxa	1.692E-04	8.460E-05	0.391	1
Firmicutes - Clostridia - Clostridiales - Lachnospiraceae - Lachnospiraceae ND3007 group	-4.230E-04	0	0.398	1
Firmicutes - Clostridia - Clostridiales - Lachnospiraceae - Coprococcus 1	-1.692E-04	8.460E-05	0.398	1
Firmicutes - Clostridia - Clostridiales - Ruminococcaceae - Faecalibacterium	7.107E-03	-1.438E-03	0.398	1
Firmicutes - Clostridia - Clostridiales - Ruminococcaceae - Ruminiclostridium 1	0	0	0.404	1
Cyanobacteria - Melainabacteria - Gastranaerophilales - gut metagenome - gut metagenome	0	0	0.408	1
Firmicutes - Clostridia - Clostridiales - Lachnospiraceae - [Eubacterium] oxidoreducens group	-8.460E-05	0	0.411	1
Proteobacteria - Gammaproteobacteria - Pasteurellales - Pasteurellaceae - Haemophilus	0	0	0.419	1
Bacteroidetes - Bacteroidia - Bacteroidales - Prevotellaceae - Prevotella 9	0	0	0.433	1
Firmicutes - Clostridia - Clostridiales - Ruminococcaceae - Ruminiclostridium 6	-8.460E-05	0	0.437	1

Firmicutes - Clostridia - Clostridiales - Lachnospiraceae - Marvinbryantia	0	0	0.452	1
Firmicutes - Clostridia - Clostridiales - Peptostreptococcaceae - Romboutsia	0	0	0.463	1
Actinobacteria - Actinobacteria - Bifidobacteriales - Bifidobacteriaceae - Bifidobacterium	-2.707E-03	-1.946E-03	0.466	1
Firmicutes - Clostridia - Clostridiales - Lachnospiraceae - [Ruminococcus] gnavus group	0	0	0.469	1
Firmicutes - Clostridia - Clostridiales - Ruminococcaceae - Ruminococcus 1	1.269E-03	1.946E-03	0.481	1
Firmicutes - Clostridia - Clostridiales - Lachnospiraceae - Roseburia	3.723E-03	1.201E-02	0.481	1
Firmicutes - Clostridia - Clostridiales - Clostridiaceae 1 - Clostridium sensu stricto 1	0	0	0.486	1
Firmicutes - Clostridia - Clostridiales - Lachnospiraceae - Butyrivibrio	0	0	0.491	1
Firmicutes - Clostridia - Clostridiales - Defluviitaleaceae - Defluviitaleaceae UCG-011	-8.460E-05	0	0.494	1
Proteobacteria - Deltaproteobacteria - Desulfovibrionales - Desulfovibrionaceae - Bilophila	-5.922E-04	-1.692E-04	0.510	1
Firmicutes - Erysipelotrichia - Erysipelotrichales - Erysipelotrichaceae - Coprobacillus	0	0	0.532	1
Firmicutes - Clostridia - Clostridiales - Lachnospiraceae - Tyzzerella	0	0	0.541	1
Firmicutes - Clostridia - Clostridiales - Lachnospiraceae - Lachnoclostridium	1.269E-03	1.861E-03	0.541	1
Bacteroidetes - Bacteroidia - Bacteroidales - Bacteroidales S24-7 group - uncultured organism	0	0	0.554	1
Firmicutes - Negativicutes - Selenomonadales - Acidaminococcaceae - Succiniclasticum	0	0	0.554	1
Firmicutes - Clostridia - Clostridiales - Ruminococcaceae - Intestinimonas	-8.460E-05	0	0.556	1
Bacteroidetes - Bacteroidia - Bacteroidales - Porphyromonadaceae - Barnesiella	0	0	0.561	1
Firmicutes - Clostridia - Clostridiales - Ruminococcaceae - Ruminococcaceae UCG-011	-3.384E-04	0	0.563	1
Firmicutes - Clostridia - Clostridiales - Christensenellaceae - Christensenellaceae R-7 group	-1.354E-03	-1.946E-03	0.573	1
Firmicutes - Clostridia - Clostridiales - Ruminococcaceae - Butyricicoccus	-6.768E-04	0	0.573	1
Firmicutes - Clostridia - Clostridiales - Family XIII - [Eubacterium] nodatum group	0	0	0.573	1
Firmicutes - Clostridia - Clostridiales - Ruminococcaceae - Ruminococcaceae UCG-010	-4.230E-04	-1.692E-04	0.576	1
Firmicutes - Clostridia - Clostridiales - Peptococcaceae - uncultured	0	0	0.579	1
Firmicutes - Clostridia - Clostridiales - Ruminococcaceae - Ruminiclostridium 9	-1.015E-03	-8.460E-04	0.589	1
Bacteroidetes - Bacteroidia - Bacteroidales - Rikenellaceae - Rikenellaceae RC9 gut group	0	0	0.591	1
Proteobacteria - Gammaproteobacteria - Enterobacteriales - Enterobacteriaceae - Hafnia-Obesumbacterium	0	0	0.591	1
Firmicutes - Clostridia - Clostridiales - Clostridiales vadinBB60 group - uncultured bacterium	0	0	0.605	1
Firmicutes - Clostridia - Clostridiales - Family XIII - Family XIII AD3011 group	-3.384E-04	-1.692E-04	0.613	1
Firmicutes - Clostridia - Clostridiales - Lachnospiraceae - Tyzzerella 3	0	0	0.616	1
Firmicutes - Clostridia - Clostridiales - Lachnospiraceae - [Eubacterium] ventriosum group	-1.692E-04	-8.460E-05	0.621	1
Cyanobacteria - Melainabacteria - Gastranaerophilales - uncultured bacterium - uncultured bacterium	0	0	0.628	1
Firmicutes - Clostridia - Clostridiales - Ruminococcaceae - Ruminococcaceae UCG-013	-1.692E-04	0	0.638	1
Proteobacteria - Betaproteobacteria - Burkholderiales - Alcaligenaceae - Sutterella	-5.076E-04	0	0.646	1
Proteobacteria - Gammaproteobacteria - Enterobacteriales - Enterobacteriaceae - Escherichia-Shigella	0	0	0.653	1
Firmicutes - Bacilli - Lactobacillales - Streptococcaceae - Streptococcus	8.460E-05	0	0.655	1
Proteobacteria - Gammaproteobacteria - Oceanospirillales - Halomonadaceae - Halomonas	0	0	0.670	1
Firmicutes - Clostridia - Clostridiales - Peptostreptococcaceae - Intestinibacter	-8.460E-05	0	0.685	1
Firmicutes - Clostridia - Clostridiales - Ruminococcaceae - Subdoligranulum	1.827E-02	8.968E-03	0.690	1
Firmicutes - Clostridia - Clostridiales - Lachnospiraceae - Lachnospiraceae UCG-006	2.538E-04	5.076E-04	0.707	1
Firmicutes - Clostridia - Clostridiales - Lachnospiraceae - [Ruminococcus] torques group	5.922E-04	8.460E-05	0.707	1
Actinobacteria - Coriobacteriia - Coriobacteriales - Coriobacteriaceae - uncultured	-8.460E-05	0	0.714	1
Firmicutes - Clostridia - Clostridiales - Lachnospiraceae - Moryella	0	0	0.718	1
Actinobacteria - Coriobacteriia - Coriobacteriales - Coriobacteriaceae - Adlercreutzia	0	0	0.722	1
Tenericutes - Mollicutes - NB1-n - uncultured organism - uncultured organism	0	0	0.722	1
Firmicutes - Clostridia - Clostridiales - Lachnospiraceae - Lachnospiraceae FCS020 group	0	0	0.724	1
Actinobacteria - Coriobacteriia - Coriobacteriales - Coriobacteriaceae - Asaccharobacter	0	0	0.729	1
Actinobacteria - Coriobacteriia - Coriobacteriales - Coriobacteriaceae - Slackia	0	0	0.733	1
Proteobacteria - Gammaproteobacteria - Pseudomonadales - Pseudomonadaceae - Pseudomonas	0	0	0.743	1
Firmicutes - Clostridia - Clostridiales - Ruminococcaceae - Hydrogenoanaerobacterium	0	0	0.748	1
Bacteroidetes - Bacteroidia - Bacteroidales - Porphyromonadaceae - uncultured	0	2.538E-04	0.760	1
Proteobacteria - Alphaproteobacteria - Rhodospirillales - Rhodospirillaceae - uncultured	0	0	0.775	1
Verrucomicrobia - Verrucomicrobiae - Verrucomicrobiales - Verrucomicrobiaceae - Akkermansia	-8.460E-05	-8.460E-04	0.778	1
Firmicutes - Clostridia - Thermoanaerobacterales - Thermoanaerobacteraceae - Gelria	0	0	0.781	1
Firmicutes - Clostridia - Clostridiales - Lachnospiraceae - [Ruminococcus] gauvreauii group	8.460E-05	0	0.785	1
Firmicutes - Clostridia - Clostridiales - Family XIII - Family XIII UCG-001	0	-1.692E-04	0.786	1
Firmicutes - Clostridia - Clostridiales - Lachnospiraceae - Lactonifactor	0	0	0.794	1
Firmicutes - Clostridia - Clostridiales - Lachnospiraceae - Lachnospiraceae NK4A136 group	1.438E-03	1.354E-03	0.796	1
Firmicutes - Clostridia - Clostridiales - Ruminococcaceae - Ruminococcaceae UCG-004	0	0	0.803	1
Lentisphaerae - Lentisphaeria - Victivallales - Victivallaceae - Victivallis	0	0	0.809	1
Firmicutes - Clostridia - Clostridiales - Ruminococcaceae - Ruminiclostridium	0	0	0.814	1
Firmicutes - Clostridia - Clostridiales - Lachnospiraceae - Fusicatenibacter	-9.306E-04	3.384E-04	0.814	1
Firmicutes - Clostridia - Clostridiales - Lachnospiraceae - Lachnospiraceae NC2004 group	-3.384E-04	8.460E-05	0.814	1
Tenericutes - Mollicutes - Mollicutes RF9 - uncultured bacterium - uncultured bacterium	0	0	0.816	1
Firmicutes - Clostridia - Clostridiales - Ruminococcaceae - Candidatus Soleaferrea	0	0	0.833	1
Firmicutes - Clostridia - Clostridiales - Lachnospiraceae - Lachnospiraceae UCG-001	8.460E-05	0	0.841	1
Firmicutes - Clostridia - Clostridiales - Christensenellaceae - uncultured	-8.460E-05	-8.460E-05	0.851	1
Firmicutes - Clostridia - Clostridiales - Ruminococcaceae - Ruminococcus 2	-5.922E-04	-7.614E-04	0.851	1
Firmicutes - Clostridia - Clostridiales - Lachnospiraceae - Lachnospira	2.115E-03	1.354E-03	0.851	1
Firmicutes - Clostridia - Clostridiales - Ruminococcaceae - Ruminococcaceae UCG-003	0	0	0.860	1
Firmicutes - Clostridia - Clostridiales - Lachnospiraceae - Lachnospiraceae UCG-003	0	0	0.860	1
Firmicutes - Clostridia - Clostridiales - Lachnospiraceae - Dorea	-9.306E-04	-1.015E-03	0.869	1
Firmicutes - Erysipelotrichia - Erysipelotrichales - Erysipelotrichaceae - Holdemania	0	0	0.877	1
Firmicutes - Clostridia - Clostridiales - Clostridiales vadinBB60 group;Ambiguous_taxa	-8.460E-05	-8.460E-05	0.878	1
Firmicutes - Clostridia - Clostridiales - Lachnospiraceae - [Eubacterium] rectale group	9.306E-04	7.614E-04	0.888	1
Firmicutes - Clostridia - Clostridiales - Ruminococcaceae - Ruminococcaceae UCG-014	0	0	0.897	1
Bacteroidetes - Bacteroidia - Bacteroidales - Rikenellaceae - Alistipes	-6.599E-03	-7.614E-04	0.906	1
Firmicutes - Clostridia - Clostridiales - Ruminococcaceae - Ruminococcaceae UCG-005	-3.384E-04	-3.384E-04	0.916	1
Firmicutes - Erysipelotrichia - Erysipelotrichales - Erysipelotrichaceae - Holdemanella	0	0	0.917	1
Firmicutes - Clostridia - Clostridiales - Lachnospiraceae - Tyzzerella 4	0	0	0.922	1
Firmicutes - Clostridia - Clostridiales - Lachnospiraceae - [Eubacterium] xylanophilum group	0	0	0.925	1

Actinobacteria - Actinobacteria - Bifidobacteriales - Bifidobacteriaceae - Gardnerella	-8.460E-05	-8.460E-05	0.925	1
Bacteroidetes - Bacteroidia - Bacteroidales - Porphyromonadaceae - Parabacteroides	-5.076E-04	-1.015E-03	0.925	1
Firmicutes - Clostridia - Clostridiales - Lachnospiraceae - Lachnospiraceae AC2044 group	0	0	0.932	1
Proteobacteria - Deltaproteobacteria - Desulfovibrionales - Desulfovibrionaceae - Desulfovibrio	0	0	0.940	1
Firmicutes - Clostridia - Clostridiales - Lachnospiraceae - Eisenbergiella	-8.460E-05	-8.460E-05	0.944	1
Proteobacteria - Gammaproteobacteria - Enterobacteriales - Enterobacteriaceae;Ambiguous_taxa	0	0	0.948	1
Proteobacteria - Betaproteobacteria - Burkholderiales - Oxalobacteraceae - Oxalobacter	0	0	0.950	1
Firmicutes - Clostridia - Clostridiales - Ruminococcaceae - uncultured	-4.230E-04	-3.299E-03	0.953	1
Firmicutes - Clostridia - Clostridiales - Ruminococcaceae - Anaerotruncus	-1.184E-03	-1.184E-03	0.963	1
Firmicutes - Clostridia - Clostridiales - Lachnospiraceae - [Eubacterium] eligens group	8.460E-04	2.538E-04	0.963	1
Firmicutes - Clostridia - Clostridiales - Lachnospiraceae - Blautia	-1.438E-03	-2.538E-04	0.981	1
Proteobacteria - Gammaproteobacteria - Enterobacteriales - Enterobacteriaceae - Kluyvera	0	0	0.984	1
Actinobacteria - Coriobacteriia - Coriobacteriales - Coriobacteriaceae - Enorma	0	0	1.000	1
Actinobacteria - Coriobacteriia - Coriobacteriales - Coriobacteriaceae - Olsenella	0	0	1.000	1
Bacteroidetes - Bacteroidia - Bacteroidales - Prevotellaceae - Paraprevotella	0	0	1.000	1
Bacteroidetes - Bacteroidia - Bacteroidales - Prevotellaceae - Prevotella 7	0	0	1.000	1
Cyanobacteria - Melainabacteria - Gastranaerophilales;Ambiguous_taxa;Ambiguous_taxa	0	0	1.000	1
Firmicutes - Bacilli - Lactobacillales - Streptococcaceae - Lactococcus	0	0	1.000	1
Firmicutes - Clostridia - Clostridiales - Lachnospiraceae - [Eubacterium] ruminantium group	0	0	1.000	1
Firmicutes - Clostridia - Clostridiales - Lachnospiraceae - Sellimonas	0	0	1.000	1
Firmicutes - Erysipelotrichia - Erysipelotrichales - Erysipelotrichaceae - Catenisphaera	0	0	1.000	1
Firmicutes - Negativicutes - Selenomonadales - Acidaminococcaceae - Phascolarctobacterium	0	0	1.000	1
Firmicutes - Negativicutes - Selenomonadales - Veillonellaceae - Allisonella	0	0	1.000	1
Firmicutes - Negativicutes - Selenomonadales - Veillonellaceae - Megamonas	0	0	1.000	1
Lentisphaerae - Lentisphaeria - Victivallales - vadinBE97 - uncultured bacterium	0	0	1.000	1

*Change in relative abundances from baseline to study end in the rosuvastatin group vs. the placebo group compared using the Mann–Whitney U test. False discovery rate (FDR) corrected *P* values (Q_{FDR}) was calculated according to Benjamini–Hochberg.

Supplementary Table S4

Supplementary Table S4. Top 20 gene functions altered by rosuvastatin.

KEGG orthology		Median change in relative abundance (x10^{-6}) rosuvastatin	placebo	*P* value	Q_{FDR}
K06236	collagen, type I/II/III/V/XI, alpha	-4.99	2.19	0.0001	0.255
K15584	nickel transport system substrate-binding protein	-7.67	0.04	0.0002	0.293
K02009	cobalt transport protein	-3.10	1.00	0.0003	0.293
K00094	galactitol-1-phosphate 5-dehydrogenase	-3.47	0.58	0.0004	0.293
K02489	two-component system, cell cycle sensor kinase an response regulator	-10.5	5.57	0.0007	0.293
K08321	putative autoinducer-2 (AI-2) aldolase	-4.53	0.84	0.0008	0.293
K00908	Ca2+/calmodulin-dependent protein kinase	-5.86	-0.91	0.0008	0.293
K03753	molybdopterin-guanine dinucleotide biosynthesis protein B	-4.74	2.73	0.0009	0.293
K13919	propanediol dehydratase medium subunit	-4.20	-0.15	0.0009	0.293
K13920	propanediol dehydratase small subunit	-3.53	-0.12	0.0009	0.293
**K14084**	**trimethylamine corrinoid protein**	**-5.39**	**3.32**	**0.0009**	**0.293**
**K05245**	**L-carnitine/gamma-butyrobetaine antiporter**	**-7.92**	**-0.60**	**0.0010**	**0.295**
**K03451**	**betaine/carnitine transporter, BCCT family**	**-44.40**	**0.83**	**0.0013**	**0.311**
K03738	aldehyde:ferredoxin oxidoreductase	-27.30	6.75	0.0013	0.311
K05881	PTS hybrid protein	-4.39	-0.03	0.0013	0.311
K01599	uroporphyrinogen decarboxylase	-35.30	21.10	0.0015	0.319
K01531	Mg2+-importing ATPase	-33.30	14.80	0.0016	0.329
**K14083**	**trimethylamine-corrinoid protein Co-methyltransferase**	**-81.30**	**-6.26**	**0.0017**	**0.331**
K10939	accessory colonization factor AcfD	-4.49	1.31	0.0019	0.331
K00263	leucine dehydrogenase	-4.78	-0.04	0.0021	0.331

Change in relative abundances from baseline to study end compared using the Mann–Whitney U test. False discovery rate (FDR) corrected *P* values (Q_{FDR}) was calculated according to Benjamini–Hochberg, on the basis of all annotated functions (Kegg Orthologs) in the dataset (n = 3506). Functions related to the choline/betaine-trimethylamine metabolic pathway in bold.

Supplementary Table S5

Supplementary Table S5. Correlations between the change in abundance of inferred genetic microbiome content and change in plasma levels of carnitine metabolites in the placebo group (*n* = 17, top), and in the rosuvastatin group (n = 16, bottom) during follow-up.

	K03451 Betaine/carnitine transporter (BCCT family)		K05020 Glycine betaine transporter		K05245 L-carnitine/ γ-butyrobetaine antiporter		K14083 Trimethylamine-corrinoid protein co-methyltransferase	
	rho	*P*	rho	*P*	rho	*P*	rho	*P*
betaine	0.397	0.115	**0.581**	**0.014**	0.387	0.125	**0.600**	**0.011**
carnitine	-0.304	0.236	0.091	0.729	-0.056	0.830	0.103	0.694
choline	0.047	0.859	0.283	0.271	-0.009	0.974	0.202	0.436
γ-butyrobetaine	0.375	0.138	**0.542**	**0.025**	0.355	0.162	**0.529**	**0.029**
TMAO*	0.048	0.855	0.163	0.532	0.085	0.747	0.193	0.459

	K03451 Betaine/carnitine transporter (BCCT family)		K05020 Glycine betaine transporter		K05245 L-carnitine/ γ-butyrobetaine antiporter		K14083 Trimethylamine-corrinoid protein co-methyltransferase	
	rho	*P*	rho	*P*	rho	*P*	rho	*P*
betaine	0.050	0.854	0.241	0.368	0.179	0.506	0.082	0.762
carnitine	0.127	0.640	-0.075	0.782	-0.040	0.884	0.021	0.940
choline	0.229	0.393	0.197	0.464	0.153	0.572	0.056	0.837
γ-butyrobetaine	-0.224	0.405	-0.132	0.625	-0.065	0.812	-0.179	0.506
TMAO*	-0.226	0.399	-0.132	0.625	-0.047	0.863	-0.159	0.557

*trimethylamine-N-oxide.

Supplementary Figures

Supplementary Fig. S1

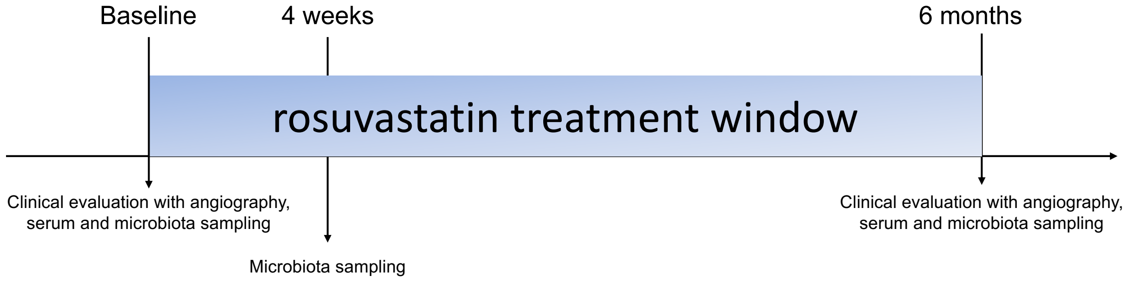

Supplementary Fig. S1. Study design.

Supplementary Fig. S2

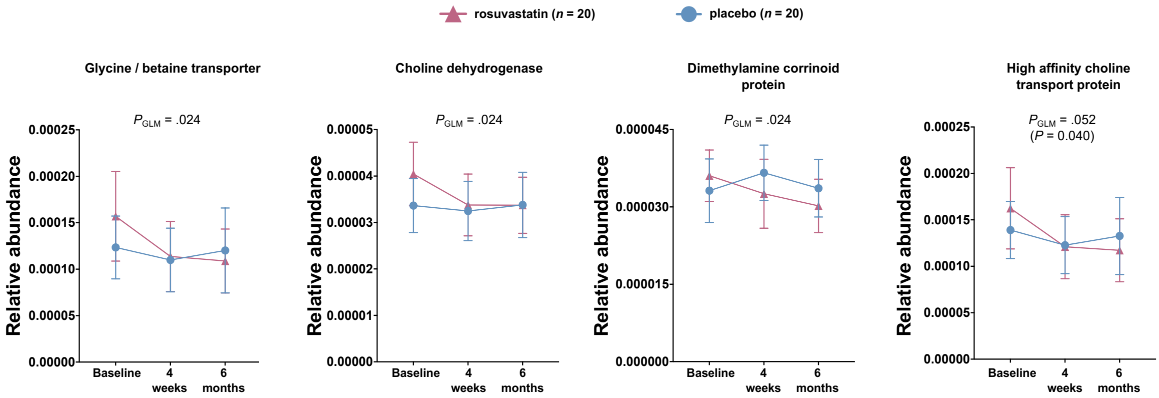

Supplementary Fig. S2. Functions (Kegg Orthologs) in the gut microbiota related to cellular transport and metabolism along the choline/betaine-trimethylamine (TMA) metabolic pathway are reduced on rosuvastatin treatment. The relative abundance of the different functions were estimated using Tax4Fun.[1] The reduction in genes coding for "high-affinity choline transport protein" in the rosuvastatin group was significant compared to the change in the placebo group when compared with the Mann-Whitney U test (P = 0.040), but did not meet the significance threshold when analyzed with repeated measures ANOVA P_{GLM} = 0.052). Data shown as mean±95%CI. Repeated measures ANOVA from baseline and study-end, denoted P_{GLM}. Values at 4 weeks missing for n = 1 in each group.

Supplementary Fig. S3

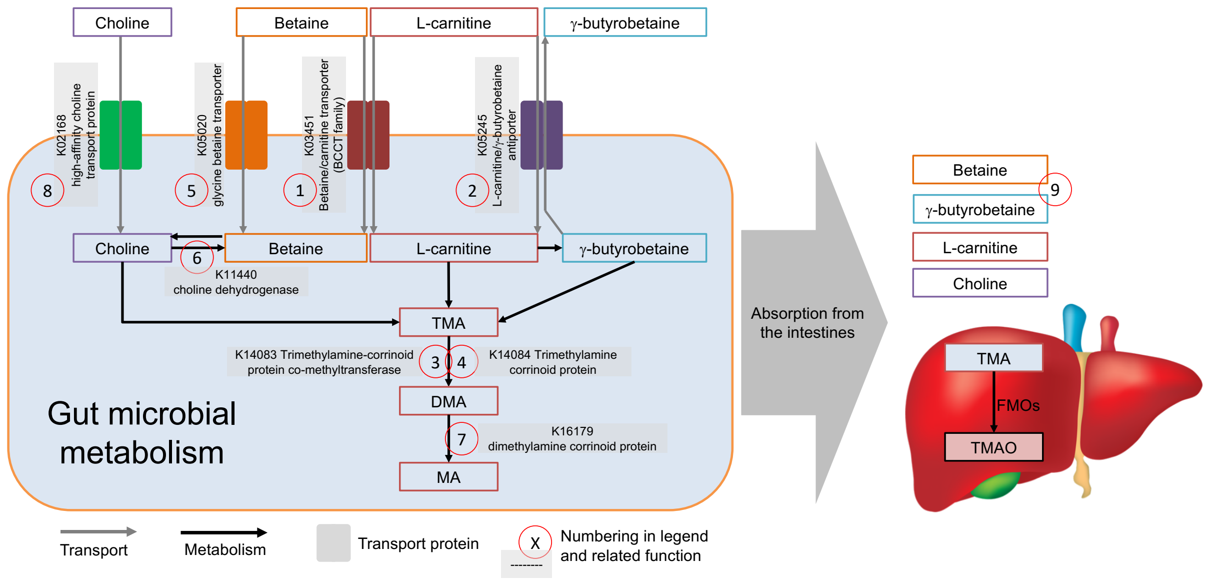

Supplementary Fig. S3. Overview of gut microbial metabolism of trimethylamine (TMA) and the precursors choline, betaine, L-carnitine and γ-butyrobetaine, and relevant results from the present study. Betaine is converted to choline, that in addition to L-carnitine and γ-butyrobetaine can be further metabolized to TMA.[2] TMA, and the other metabolites are absorbed into the human circulation were TMA is metabolized to trimethylamine-N-oxide in the liver by hepatic flavin monooxygenases (FMOs). In the present study, rosuvastatin treatment induced a reduction in several microbial genes related to transport and metabolism of TMA and its precursors, compared to the placebo group, including: (1) Betaine/carnitine transporter (BCCT family)[3] (K03451); (2) L-carnitine/γ-butyrobetaine antiporter[4] (K05245); (3) Trimethylamine-corrinoid protein co-methyltransferase (K14083) and (4) Trimethylamine corrinoid protein (K14084), both involved in the degradation of TMA to dimethylamine (DMA); (5) glycine betaine transporter (K05020)[5]; (6) choline dehydrogenase (K11440) is involved in the biosynthesis of betaine from choline; (7) dimethylamine corrinoid protein (K16179) is involved in degradation of DMA to methylamine (MA); (8) High-affinity choline transport protein (K02168). We also detected an increase in both betaine and γ-butyrobetaine in plasma in the rosuvastatin group compared to the placebo group (9), in addition to a trend towards increase of both choline and carnitine in plasma in the rosuvastatin group compared to placebo (P_{GLM} = 0.113 and 0.097, respectively). However, these metabolites, in contrast to TMA, also have endogenous sources in humans.[6,7] Taken together, our data suggest that rosuvastatin treatment could interfere with the ability of the gut microbiota to metabolize and transport betaine and related metabolites, with a corresponding increase in the serum levels of related metabolites. Red numbers in parenthesis in the legend corresponds to the red-circled numbers in the figure.

Supplementary References

1. Aßhauer, K. P., Wemheuer, B., Daniel, R. & Meinicke, P. Tax4Fun: predicting functional profiles from metagenomic 16S rRNA data. *Bioinformatics* **31**, 2882–4 (2015). https://doi.org/10.1093/bioinformatics/btv287
2. Claus, S. P. Mammalian-microbial cometabolism of L-carnitine in the context of atherosclerosis. *Cell Metab.* **20**, 699–700 (2014). https://doi.org/10.1016/j.cmet.2014.10.014
3. Ziegler, C., Bremer, E. & Krämer, R. The BCCT family of carriers: from physiology to crystal structure. *Mol. Microbiol.* **78**, 13–34 (2010). https://doi.org/10.1111/j.1365-2958.2010.07332.x
4. Schulze, S., Köster, S., Geldmacher, U., Terwisscha Van Scheltinga, A. C. & Kühlbrandt, W. Structural basis of Na+-independent and cooperative substrate/product antiport in CaiT. *Nature* **467**, 233–236 (2010). https://doi.org/10.1038/nature09310
5. Kappes, R. M., Kempf, B. & Bremer, E. Three transport systems for the osmoprotectant glycine betaine operate in Bacillus subtilis: characterization of OpuD. *J. Bacteriol.* **178**, 5071–9 (1996). https://doi.org/10.1128/jb.178.17.5071-5079.1996
6. Skagen, K. *et al.* The Carnitine-butyrobetaine-trimethylamine-N-oxide pathway and its association with cardiovascular mortality in patients with carotid atherosclerosis. *Atherosclerosis* **247**, 64–9 (2016). https://doi.org/10.1016/j.atherosclerosis.2016.01.033
7. Rajaie, S. & Esmaillzadeh, A. Dietary choline and betaine intakes and risk of cardiovascular diseases: review of epidemiological evidence. *ARYA Atheroscler.* **7**, 78–86 (2011). Available at http://arya.mui.ac.ir/index.php/arya/article/view/250
